# Migration of implanted markers for image‐guided lung tumor stereotactic ablative radiotherapy

**DOI:** 10.1120/jacmp.v14i2.4046

**Published:** 2013-03-04

**Authors:** Julian C. Hong, Neville C.W. Eclov, Yao Yu, Aarti K. Rao, Sonja Dieterich, Quynh‐Thu Le, Maximilian Diehn, Daniel Y. Sze, Billy W. Loo, Nishita Kothary, Peter G. Maxim

**Affiliations:** ^1^ Department of Radiation Oncology School of Medicine, Stanford University Stanford CA; ^2^ Stanford Cancer Institute, School of Medicine, Stanford University Stanford CA; ^3^ Institute for Stem Cell Biology and Regenerative Medicine School of Medicine, Stanford University Stanford CA; ^4^ Division of Interventional Radiology School of Medicine, Stanford University Stanford CA; ^5^ Department of Radiology School of Medicine, Stanford University Stanford CA; ^6^ University of Wisconsin School of Medicine and Public Health Madison WI; ^7^ Committee on Medical Physics University of Chicago Chicago IL; ^8^ Department of Radiation Oncology Sacramento CA USA; ^9^ University of California Davis School of Medicine Sacramento CA USA

**Keywords:** implanted fiducial marker migration, stereotactic ablative radiotherapy (SABR), stereotactic body radiotherapy (SBRT), endovascular embolization coils, gold seed fiducial markers

## Abstract

The purpose of this study was to quantify postimplantation migration of percutaneously implanted cylindrical gold seeds (“seeds”) and platinum endovascular embolization coils (“coils”) for tumor tracking in pulmonary stereotactic ablative radiotherapy (SABR). We retrospectively analyzed the migration of markers in 32 consecutive patients with computed tomography scans postimplantation and at simulation. We implanted 147 markers (59 seeds, 88 coils) in or around 34 pulmonary tumors over 32 procedures, with one lesion implanted twice. Marker coordinates were rigidly aligned by minimizing fiducial registration error (FRE), the root mean square of the differences in marker locations for each tumor between scans. To also evaluate whether single markers were responsible for most migration, we aligned with and without the outlier causing the largest FRE increase per tumor. We applied the resultant transformation to all markers. We evaluated migration of individual markers and FRE of each group. Median scan interval was 8 days. Median individual marker migration was 1.28 mm (interquartile range [IQR] 0.78−2.63 mm). Median lesion FRE was 1.56 mm (IQR 0.92−2.95 mm). Outlier identification yielded 1.03 mm median migration (IQR 0.52−2.21 mm) and 1.97 mm median FRE (IQR 1.44−4.32 mm). Outliers caused a mean and median shift in the centroid of 1.22 and 0.80 mm (95th percentile 2.52 mm). Seeds and coils had no statistically significant difference. Univariate analysis suggested no correlation of migration with the number of markers, contact with the chest wall, or time elapsed. Marker migration between implantation and simulation is limited and unlikely to cause geometric miss during tracking.

PACS number: 87.57.N‐; 87.57.nm; 87.53.Ly

## I. INTRODUCTION

Stereotactic ablative radiotherapy (SABR), also known as stereotactic body radiation therapy (SBRT), has been an important development in radiation therapy for small malignant lung tumors.^(^ ^1^ ^,^ ^2^ ^)^ SABR involves the precise delivery of high doses of conformal radiation over fewer fractions to ablate lesions while sparing surrounding healthy tissue. The use of image‐guided radiation therapy (IGRT) is also critical for pulmonary lesions due to their motion over the respiratory cycle and unfixed relationship with the skeletal anatomy. However, because pulmonary lesions in soft tissue lack high radiopaque contrast with planar imaging, IGRT of the lung remains challenging. Volumetric computerized tomography (CT) is often limited in its ability to dynamically track tumors due to insufficient temporal resolution. In situations where significant motion demands the use of strategies like tracking or respiratory gating, the use of radiopaque fiducial markers to enhance tumor localization is a useful strategy.

The use of surrogates was originally pioneered for intracranial radiation therapy in lieu of a much more invasive rigid skull fixation.^(^ ^3^ ^)^ In a continuing effort to reduce invasiveness, interventional radiological implantation of round gold markers facilitating the use of real‐time stereoscopic fluoroscopy in automated image‐guided respiratory‐gated radiation therapy was later developed in Japan.^(^ ^4^ ^,^ ^5^ ^)^ These principles were soon after applied to a commercial system in the form of dynamic tumor tracking using automated detection and trajectory modeling of implanted gold markers (CyberKnife, Accuray, Inc., Sunnyvale, CA). The CyberKnife uses periodic stereoscopic X‐ray imaging in combination with continuous optical imaging of light‐emitting diode markers placed on the external body surface to facilitate tracking of lesions.^(^ ^6^ ^,^ ^7^ ^)^

A comparison between platinum vascular embolization coils (“coils”) and gold cylindrical seeds (“seeds”) as fiducial markers was previously reported.^(^ ^8^ ^)^ This study showed that coils had superior retention in tissue with minimal complications, such as pneumothorax, and mild CT artifacts. Loss of markers, when it happened, was found to occur immediately postimplantation. Imaging studies at post‐treatment follow‐up indicated no long‐term marker loss. While failure of marker retention will compromise the accuracy of tumor tracking, marker migration over the course of time could potentially result in the misdirection of the treatment beam and thus in underdosage of the tumor and/or in overdosage of the surrounding healthy tissue. A previous study by Kupelian et al.^(^ ^9^ ^)^ investigated fiducial stability in a 23‐patient cohort relative to gross tumor volume (GTV), finding that marker variation was minimal. However, these findings were limited both by changing tumor anatomy and user variability in defining the GTV. It is therefore the aim of this study to propose a novel alternative method to quantify the postimplantation migration of percutaneously placed fiducial markers for tumor tracking in SABR of pulmonary tumors. Secondarily, we intend to compare the migration of seeds and coils. We propose to apply the fiducial registration error (FRE), a common metric used to quantify image misalignment between volumetric images, to assess marker migration on CT scans at different time points, compare migration of seeds and coils, and identify potentially predictive attributes of lesions that may impact marker migration (number of markers tracked, time elapsed between scans, and lesion distance to the chest wall).

## II. MATERIALS AND METHODS

### A. Patient characteristics

This retrospective study was conducted with the approval of the institutional review board. We analyzed marker migration in 32 consecutive patients who underwent fiducial marker placement in preparation for radiation therapy of pulmonary tumors and who had CT after placement.

Thirty‐two patients (13 men, 19 women) underwent 32 implantation procedures, with placement of 147 fiducial markers in or around 34 lung lesions. One lesion was implanted twice during a single procedure. Of these, 59 of the markers were seeds (placed between January 2004 and September 2008) and 88 were coils (placed between June 2008 and June 2009). Tumors were implanted with either seeds or coils alone. Of these lesions, 32 (94%) were treated by SABR, 31 using the CyberKnife system, and one using the Trilogy system (Varian Medical Systems, Palo Alto, CA). Two additional lesions were treated with conventionally fractionated IGRT using the Trilogy system. Additional patient and tumor characteristics are summarized in Table 1.

**Table 1:** Patient and tumor characteristics.

*Parameter*	*Value*
Total # of Patients	32
Mean patient age	70
Gender	
Men	13
Women	19
Tumor Histology	
NSCLC (early stage)	28
Metastatasis from other primary site	4
Total # of Lesions	34
Treatment platform	
CyberKnife	31
Trilogy	3
Treatment Modality	
SABR	32
Conventionally fractionated RT	2

### B. Placement of fiducial markers

As described in prior literature, fiducial markers were implanted under CT fluoroscopy guidance.^(^ ^8^ ^,^ ^10^ ^)^ Smooth cylindrical gold seed markers (Alpha‐Omega Services, Bellflower, CA) and platinum endovascular embolization coils (VortX 18 Vascular Occlusion Coil, Boston Scientific Corp., Natick, MA) were used as fiducial markers. Seeds were 0.8 mm in diameter by 5 mm in length and were delivered via 19‐gauge thin‐wall coaxial introducer needle. Coils were platinum wires insinuated with polyester fibers stored straight in an inducer, which, upon delivery, coiled into spherical shapes with a diameter of 3 mm. They were delivered percutaneously through a 19‐gauge (n=15) or 22‐gauge (n=4) coaxial needle under CT guidance using a similar technique as with the seeds.

### C. Ct imaging

CT scans were taken immediately following implantation of markers and at the time of simulation. Postimplantation scans were acquired during free breathing in helical mode with a slice thickness of 5 mm. Treatment planning scans were 4D scans performed on the GE Discovery ST multislice PET/CT scanner (General Electric Medical Systems, Waukesha, WI) in cine mode. During the CT scan, patient respiratory traces were acquired using the Real‐Time Position Management system (RPM) (Varian Medical Systems, Palo Alto, CA) with the external markers placed on the upper abdomen. Data were acquired at each bed position for a cine duration that was set to 1 second (s) longer than the average observed respiratory period for each patient. Scan parameters were set as follows: 0.5 s gantry rotation, 0.45 s cine interval, and 2.5 mm slice thickness. Each image reconstruction took 360 degrees of projection data. The breath‐hold scans were acquired in helical mode with 1.25 mm slice thickness.

### D. Data analysis

**Figure 1:**
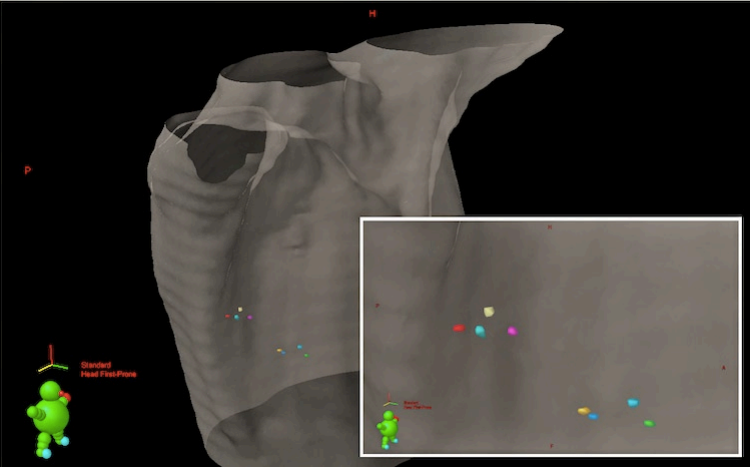
CT scan with markers contoured (with inset). Markers were implanted around two separate right lung tumors.

**Figure 2:**
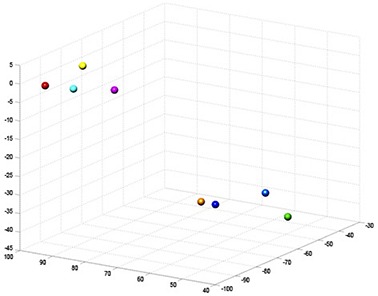
CT coordinates from the patient shown in Fig. 1 rendered in MATLAB for analysis. Each sphere represents a marker in Fig. 1.

Patients' CT scans were analyzed in the context of this study. Those fiducial markers that were lost from the tumor prior to the postimplantation CT scan were not included in this analysis. Fiducial markers were counted and compared to the recorded number of markers implanted in procedure reports. The MIM software suite (MIM Software Inc., Cleveland, OH) was used to contour the fiducial markers for each patient's postimplantation and simulation CT scans (Fig. 1). Each marker was represented as a point at the coordinates of its center of mass calculated by MIM (Fig. 2).

Using these points, custom scripts written with the MATLAB mathematical package (The MathWorks Inc., Natick, MA) were used to compute the FRE, defined as the root mean square (RMS) of the errors between a marker's coordinates at each time point. To eliminate the effects of lesion motion over the respiratory cycle and patient positioning, an optimal rigid transformation to minimize the FRE between marker coordinates was determined first.

In order to establish this single coordinate system, marker coordinate sets at both postimplantation and simulation were translated such that both centroids were aligned at the origin (0,0,0). The rotational component of the rigid transformation, the rotational matrix, was calculated by solving the orthogonal Procrustes problem for matrices representing the coordinates of the implanted markers at postimplantation and simulation, respectively. The result yields the matrix that generates the minimum FRE.^(^ ^11^ ^)^ This minimum FRE represents the remaining nonrigid migration.

To do so, we first calculated the weighted fiducial covariance matrix, H. For our purposes, each fiducial was weighted equally:

(1) $$H={\text{AB}}^{T}$$

where *A* and *B* represent the fiducial coordinate matrices of the postimplantation and treatment planning scans aligned at the origin. The singular value decomposition of *H* was calculated:

(2) $$H={\mathrm{U\Lambda V}}^{T}$$

where $U^{t}U=V^{t}V=I$, the identity matrix, $\Lambda =\text{diag}\left(\lambda_{1},\lambda_{2},\lambda_{3}\right)$, and $\lambda_{1}\geq \lambda_{2}\geq \lambda_{3}\geq 0$. The orthogonal rotation matrix *R* was then computed, providing the transformation matrix for the rigid rotation:

(3) $$R=\text{Vdiag}(1,1,\text{det}(\text{VU}))U^{T}$$

**Figure 3:**
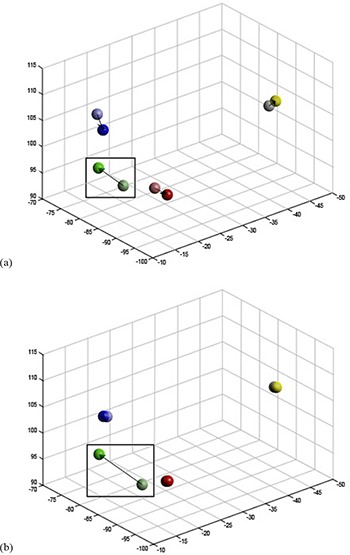
Sample patient marker coordinates (a) aligned including all markers. Colors are representative of a single marker over postimplantation (lighter) and simulation (darker) CT scans. Migration determined with this alignment method is represented by the arrow. The alignment is less accurate than that when considering the outlier. Alignment with outlier identification (b). In comparison to (a), the outlier identification excludes the largest outlier (green; square) from the alignment. In this example, the remaining markers are significantly better aligned with their counterparts and show significantly less migration, suggesting that a single marker was primarily responsible for the observed registration error prior to outlier identification.

The resulting mapping and a cocentroidal translation were applied to the postimplantation marker coordinates to do a “best transformation” (Fig. 3). The FRE was then computed comparing these transformed coordinates to those of the treatment plan. Individual errors between markers (representing “marker migration”) were determined, as well.

### E. Outlier identification

**Figure 4:**
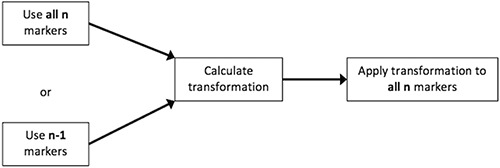
Algorithm for alignment and calculation for FRE. Calculation of the transformation matrix can be done including all markers or excluding the outlier. However, the transformation is applied to all markers to calculate FRE.

Occasionally, error from the large migration of a single marker can generate inaccuracies in our alignment by distributing the error across all four of the markers (Fig. 3(a)). To appropriately characterize and quantify migration, this effect needs to be captured. Thus, we calculated the optimal (lowest FRE) transformation for each lesion by iterating through the exclusion of each of the markers in a lesion and calculating the FRE for the remaining (n−1) markers (i.e., for all sets of three of four markers) using the calculation methods in Fig. 4. The optimal transformation was found to be the solution in which the excluded marker was responsible for the greatest increase in FRE. This marker was labeled as the outlier. This transformation was then applied to all n markers, including the outlier (Fig. 3(b)), to provide a more accurate fit. From this transformation, the total FRE was calculated to determine the overall magnitude of migration. The result is a better alignment of the remaining (n−1) markers, and error is appropriately attributed to the outlier.

**Figure 5:**
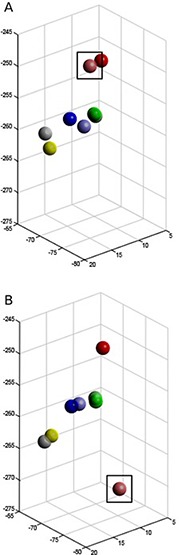
Exclusions from outlier identification. In cases where n−1 markers are collinear, it is possible for the optimal solution to be a rotation about the shared axis. In these cases, this can rotate the “outlier” marker (square) to a distant position, inappropriately attributing migration to a single marker. The two cases where this occurred were manually verified to be inconsistent and excluded.

Of note, lesions that were implanted with only three markers were excluded from the ‘outlier identification’ calculation (eight lesions). While registration and alignment are possible with two markers, three are necessary to completely define the rotation problem. Registration with only two points does not generate unique solutions, as these points define a line or axis, which could include any rotation about it. Similarly and less commonly, it is possible for three markers in a set of four to be near collinear, generating incorrect solutions. In such cases, one can imagine a scenario where the collinear markers have experienced minimal migration that would be better aligned through rotation about a line rather than translation. When identifying and excluding the marker that is not collinear, the FRE‐minimizing rotation would result in simple rotation about the line on which the markers are located. This can lead to placement of the “outlying” fiducial in positions that are farther than they should be, disproportionately attributing error to single markers (Fig. 5). Thus, we screened for such situations and two lesions were found to have a disparity of greater than 30° between the transformation calculated with the outlier exclusion method and inclusion of all markers. We manually reviewed these images to verify that this transformation was inaccurate and excluded from our outlier identification analysis. Therefore, 25 sets of fiducials were analyzed using this method.

### F. Impact on center of mass

The use of outlier identification also facilitates the calculation of a center of mass shift due to the outlier. The calculation of the rigid transformation assumes that the centroid for the (n−1) nonoutlier markers remains constant. Reincorporation of the outlier shifts this center of mass. The adjusted centroid was calculated with all n fiducials at postimplantation and at simulation, and the shift was calculated as the simple Euclidian distance between these centroids.

### G. Comparison

FRE and error output from the transformation strategies were compared between the two marker types. Marker migration was compared between seeds and coils using a two‐tailed t‐test. Furthermore, we performed univariate analyses of potentially predictive variables for FRE using two‐tailed t‐tests, dividing each variable at the median: number of markers tracked (>four markers), time elapsed between scans (>four eight days), and tumor contact with the pleural surface (> 0 cm). Statistics were performed in SAS 9.3 (SAS Institute Inc., Cary, NC).

## III. RESULTS

Marker retention characteristics are summarized in Table 2. A median of 4 seeds or 4 coils were implanted per tumor (ranges 3–5 seeds, 3–6 coils). Results were generally reflective of previously reported findings in the literature.^(^ ^8^ ^)^ Compared to seeds, coils were retained at a higher rate (100% vs. 93.2%). Median time between implantation and simulation was eight days (range 5–248 days). This high range was driven by four patients (range 42–248 days) with delayed simulation caused by failed biopsy, inpatient admission, and intervening radiation treatments. Excluding these patients, the mean time elapsed between implantation and simulation was 9.6 days.

**Table 2:** Comparison of seed and coil fiducial markers.

	*Total*	*Seeds*	*Coils*
Total placed	147	59	88
Total retained	143	55	88
Retention rate	97.3%	93.2%	100%
Total # of implantation procedures^a^	32	13	18
Lesions initially implanted	34	14	20
Lesions requiring additional markers	1	1	0
Replacement with seeds	0	0	N/A
Replacement with coils	1	1	N/A
Lesions analyzed by odd one out	25	7	18
Fiducial Markers per Lesion			
Median number placed	4	4	4
Mean number placed	4.2	4.2 (range 3–5)	4.2 (range 3–6)
Lesion Distance to Pleural Surface			
Median distance (cm)	0	0	0
Mean distance (cm)	0.42	0.31	0.50
	(range 0–2.5)	(range 0–1.9)	(range 0–2.5)
Time Between Scans			
Median time (days)	8	7.5	9
Mean time (days)	20.74	25.36	17.67

a One patient who had both seeds and coils implanted in one procedure and therefore is not assigned a single marker type.

Table 3 summarizes the errors, representing migration, between the postimplantation and treatment‐planning CT scans with and without outlier identification. Considering all markers on a per‐lesion basis without outlier identification, the mean FRE was 2.06 mm and median FRE was 1.56 mm. Interquartile range (IQR) of FRE was 0.92–2.95 mm. Seeds and coils showed comparable migration between scans, with mean FRE of 2.08 mm and 2.04 mm, respectively (p=0.932). Considering each individual marker, the mean and median migration was 1.95 mm and 1.28 mm. This metric was also comparable between the different markers, with seeds having a mean migration of 1.86 mm and coils 1.99 mm (p=0.704).

**Table 3:** FRE and individual marker migration (mm).

	*Overall*		*Seeds*		*Coils*	
	*Median IQR*	*Mean SD*	*Median IQR*	*Mean SD*	*Median IQR*	*Mean SD*
Standard Method						
FRE	1.56	2.06	1.86	2.08	1.34	2.04
	0.92–2.95	1.52	0.97–2.95	1.28	0.95–3.05	1.69
Marker migration	1.28	1.95	1.50	1.86,	1.22	1.99
	0.78–2.63	1.72	0.75–2.62	1.41	0.86–2.65	1.90
Outlier Identification						
FRE (all)	1.97	2.79	1.66	2.52	2.06	2.89
	0.44–4.32	2.06	1.18–3.78	1.81	1.48–3.97	2.19
FRE (without outlier)	0.88	1.06	0.50	0.84	0.89	1.15
	0.48–1.29	0.73	0.48–1.17	0.57	0.63–1.37	0.78
Marker migration (all)	1.03	2.01	0.69	1.71	1.20	2.13
	0.52–2.21	2.89	0.37–1.60	2.50	0.62–2.36	3.03
Outlier migration	3.42	5.47	3.22	5.09	3.96	5.62
	2.81–9.00	4.47	2.39–8.07	3.71	2.79–7.73	4.82
Nonoutlier migration^a^	0.81	1.01	0.63	0.76	0.94	1.11
	0.44–1.25	0.78	0.33–0.89	0.62	0.52–1.31	0.82

a Only migration for nonoutlier markers showed a statistically significant difference between seeds and coils (p=0.038). Overall, seeds and coils were comparable, suggesting similar amount of migration. Calculations for comparing between lesions, as well as between individual markers, yielded similar results.

Roll, pitch, and yaw calculated from the transformations were mean $-0.05^{0}$, $4.02^{0}$, and $-17.85^{0}$ and median $-0.21^{0}$, $-1.21^{0}$, and $-6.03^{0}$, respectively. Isolating the impact of outliers by calculating the transformation with outlier identification, the mean FRE considering all markers for a lesion was 2.79 mm. Seeds and coils showed no statistically significant difference, with mean tumor FRE of 2.52 and 2.89 mm, respectively (p=0.667). Individual marker migration showed the same trend; seeds were comparable to coils (1.71, 2.13 mm, p=0.450). These results are greater than those using the baseline calculation, since errors originally spread across all n fiducials are now appropriately attributed to the outlier fiducial. Excluding the outlier in the FRE calculation showed a trend that seeds resulted in less migration than coils, with a mean FRE of 0.84 and 1.15 mm, respectively (p=0.280). Nonoutlier individual marker migration indicated a significant difference between seeds and coils, with means of 0.76 and 1.11 mm, respectively (p=0.038). For outlier exclusion, roll, pitch, and yaw calculated from the transformations were mean $10.42^{0}$, $-3.35^{0}$, and $-0.45^{0}$ and median $1.47^{0}$, $-0.27^{0}$, and $1.10^{0}$, respectively.

**Figure 6:**
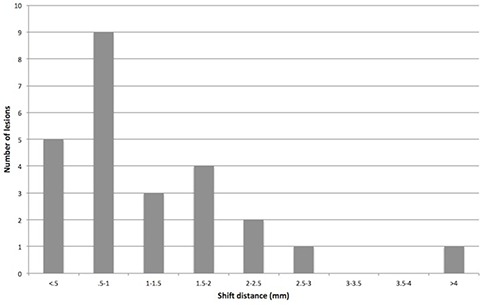
Histogram of center of mass shifts calculated when reincorporating outliers. Such shifts could be considered in planning a margin to cover potential marker migration and other sources of error.

The center of mass shifted on average 1.12 mm and a median of 0.80 mm for seeds, 1.26 mm and 0.81 mm for coils (p=0.75), and 1.22 mm and 0.80 mm overall. The maximum shift seen was 4.32 mm, the interquartile range was 0.60–1.80 mm, and the 95th percentile was 2.52 mm (Fig. 6).

The tumors in this series were predominantly peripherally located. The distance to the chest wall was not significantly different between marker types, with seeds averaging 0.31 cm and coils 0.50 cm (p=0.51). Univariate analysis (Table 4) revealed a potential trend in increased FRE with increased time between scans by standard approach and by outlier identification (p=0.161, 0.250), but findings were not statistically significant. The number of markers did not correlate with FRE (p=0.431, 0.368). For these peripheral tumors, contact with the chest wall did not predict for an increased FRE (p=0.685, 0.932).

**Table 4:** FRE by variable (mm).

	*n*	*Mean*	*SD*	p
By Standard Method				
Number of markers				
≤4	25	1.98	1.27	0.431
>4	10	2.46	2.03	
Tumor distance from chest wall				
>0 cm	12	1.92	1.19	0.685
0 cm	23	2.13	1.68	
Time elapsed between scans				
≤ 8 days	21	1.72	1.05	0.161
>8 days	14	2.56	1.97	
By Outlier Identification				
Number of markers				
≤4	15	1.90	1.27	0.368
>4	10	2.46	2.03	
Tumor distance from chest wall				
>0 cm	9	2.13	1.29	0.932
0 cm	16	2.12	1.80	
Time elapsed between scans				
≤ 8 days	15	1.71	1.00	0.250
>8	10	2.75	2.14	

## IV. DISCUSSION

Prior studies have discussed the importance of marker retention in reducing the number of markers placed to improve localization and planning.^(^ ^8^ ^)^ While retention evaluates the markers on a larger scale, migration analysis gives a more detailed insight into the accuracy of the locations specified by these surrogates.

Kupelian et al.^(^ ^9^ ^)^ previously presented data with a manual approach utilizing comparison with the GTV centroid as a point of reference. Our method allows elimination of large‐scale contouring of a rapidly changing lesion, potentially eliminating sources of variability and allowing higher throughput analysis. Other previous studies of marker migration have focused primarily on similar problems in the prostate and other sites subject to lesser degrees of respiratory motion. These primarily used Euclidian distances^(^ ^12^ ^,^ ^13^ ^)^ and comparison to the center of mass of the target organ.^(^ ^14^ ^)^ While the distance to the center of mass as a measure for marker migration is suitable where the target moves in conjunction with a well‐defined larger organ, this approach may be limited for targets that move within the larger organ — like pulmonary tumors — as the center of mass will change its location as a consequence of respiratory motion. Though the distance between markers gives a rough yet valid assessment of migration, our method uses a well‐defined metric in imaging analysis, which has been commonly and accurately applied to identifying the location of markers used in robotic surgery.^(^ ^15^ ^–^ ^17^ ^)^

This study gives insight into the characteristics of platinum endovascular embolization coils and cylindrical gold seeds as fiducial markers. Our results show that markers placed in peripheral lesions exhibit subtle migration. By various metrics, migration FRE between the time of implantation and planning simulation appears to be limited to less than 2 mm and potentially as low as around 0.5 mm for both seeds and coils. Generally, this suggests that regardless of marker type, markers that have been successfully implanted will be limited in their migration. This verifies the general reliability of markers as surrogates for tumor tracking.

The FRE between postimplantation and treatment planning scans showed limited migration when generated with and without outlier identification. Considering all markers during alignment, FRE and marker error metrics suggest comparably low migration among both marker types. However, these results may even overestimate the amount of migration occurring. Outlier identification gives insight into the distribution and migration patterns within sets of markers. Our results show that migration may be attributable to the migration of rare or single outlier markers, rather than all markers.

Tracking systems such as the CyberKnife Synchrony (CyberKnife, Accuray, Inc.) base targeting on the center of mass. Therefore, the clinical consequences of marker migration may best be evaluated by how the markers shift this point. Due to the assumptions that are made in our calculations, the outlier identification method is necessary to evaluate this shift. Assuming nonoutlier markers maintain a constant center of mass, this migration is fairly limited, with 95% of our data falling below 2.52 mm. Thus, this would be an acceptable margin to include in treatment planning.

In our calculations, we attributed all nonrigid transformations not eliminated by the transformation to marker motion, which allowed us to account for gross motion of lesions. Even if small, deformations of the lesions and the surrounding parenchyma could potentially alter the results of our analysis. Although Wu et al.^(^ ^18^ ^)^ previously reported that lung tumors are primarily subject to translation rather than rotation and deformation, it is possible that some of the apparent migration may be due to deformation from breathing.

The resolution of the postimplantation scan is also a limitation, as a 5 mm slice thickness could limit our ability to resolve migration that is smaller. The maximum detection error would occur for seeds oriented parallel to the axial plane exactly between two slices, 2.5 mm. Coils would be subject to less error, as their 3 mm diameter would result in detection error of 1 mm in each direction. Given that this exceeds the vast majority of calculated FRE, it is possible that our detected migration may simply be the result of detection error.

Furthermore, our study was also limited by the different breathing phases of the two scans — free‐breathing and expiration breath‐hold. These two scans could potentially give differently arranged coordinates for markers that are in reality in the same location. This would introduce additional error to the data misrepresented as migration. However, while all three of these limitations reduce our accuracy, they also suggest that our calculations may exceed the true migration. Thus, we can consider our results worst‐case data, furthering the point that marker migration is limited.

While this study quantified the extent of marker migration between postimplantation and simulation, a logical future study would analyze marker migration during the course of radiotherapy. Marker migration can be evaluated via daily or weekly cone‐beam CT by comparing the daily or weekly position of the markers to the initial position determined during simulation. This would allow a more direct analysis for marker migration during the course of treatment. We believe that this analysis would yield similar results given the length of time elapsed for patients in our cohort. For patients receiving SABR, the time period of treatment with fewer fractions is fairly condensed and hence would be comparable to the interval for our patients in this study.

## V. CONCLUSIONS

In summary, we have demonstrated that marker migration between the time of implantation and simulation about a week later is minimal and, although initial retention is different between seeds and coils, once retained, migration is similar and small in either case. While not included in our analysis, some of the apparent migration may be due to deformation from breathing. In this case, the presented data represent the worst‐case scenario. Our data justify CT simulation immediately after implantation. Although there may be other logistical reasons to wait, such as pneumothorax or hemorrhage, it may not be necessary to wait a week for markers to “settle”.
